# Glutathione-related genetic polymorphisms are associated with mercury retention and nephrotoxicity in gold-mining settings of a Colombian population

**DOI:** 10.1038/s41598-021-88137-3

**Published:** 2021-04-22

**Authors:** Olga Marcela Medina Pérez, Oscar Flórez-Vargas, Giovanna Rincón Cruz, Fernando Rondón González, Linda Rocha Muñoz, Luz Helena Sánchez Rodríguez

**Affiliations:** 1 Departamento de Ciencias Básicas, Universidad Industrial de Santander, Bucaramanga, Colombia; 2 Grupo de Inmunología y Epidemiología Molecular, Escuela de Microbiología, Universidad Industrial de Santander, Carrera 32 No. 29-31; Building Roberto Serpa, Floor 5, Office 5, Bucaramanga, Colombia; 3 Grupo de Investigación en Microbiología y Genética, Escuela de Biología, Universidad Industrial de Santander, Bucaramanga, Colombia; 4 Grupo de Investigación CienciaUDES, Universidad de Santander, Bucaramanga, Colombia; 5 Laboratorio de Toxicología Ambiental y Toxicogenética, Universidad Industrial de Santander, Bucaramanga, Colombia; 6 Laboratory of Translational Genomics, Division of Cancer Epidemiology and Genetics, National Cancer Institute, National Institutes of Health, Bethesda, USA

**Keywords:** Genetics, Health occupations, Nephrology

## Abstract

Mercury (Hg) vapor can produce kidney injury, where the proximal tubule region of the nephron is the main target of the Hg-induced oxidative stress. Hg is eliminated from the body as a glutathione conjugate. Thus, single nucleotide polymorphisms (SNPs) in glutathione-related genes might modulate the negative impact of this metal on the kidneys. Glutathione-related SNPs were tested for association with levels of Hg and renal function biomarkers between occupationally exposed (n = 160) and non-exposed subjects (n = 121). SNPs were genotyped by TaqMan assays in genomic DNA samples. Total mercury concentration was measured in blood, urine and hair samples. Regression analyses were performed to estimate the effects of SNPs on quantitative traits. Alleles *GCLM* rs41303970-T and *GSTP1* rs4147581-C were significantly overrepresented in the exposed compared with the non-exposed group (P < 0.01). We found significant associations for *GCLM* rs41303970-T with higher urinary clearance rate of Hg (β = 0.062, P = 0.047), whereas *GCLC* rs1555903-C was associated with lower levels of estimated glomerular filtration rate in the non-exposed group (eGFR, β = − 3.22, *P* = 0.008) and beta-2-microglobulin in the exposed group (β-2MCG, β = − 19.32, *P* = 0.02). A SNP-SNP interaction analysis showed significant epistasis between *GSTA1* rs3957356-C and *GSS* rs3761144-G with higher urinary levels of Hg in the exposed (β = 0.13, *P* = 0.04) but not in the non-exposed group. Our results suggest that SNPs in glutathione-related genes could modulate the pathogenesis of Hg nephrotoxicity in our study population by modulating glutathione concentrations in individuals occupationally exposed to this heavy metal.

## Introduction

Small-scale mining operations often use amalgamation with mercury (Hg) to recover gold; Hg is then vaporized by heating the amalgam^1^. This activity is the largest anthropogenic source of Hg pollution worldwide^2^, with Colombia being one of the main per capita Hg polluters in the world^3,4^. Inhalation of elemental Hg (Hg^0^) vapors released from burning amalgam has harmful effects on the kidneys, as it is converted to inorganic Hg (Hg1+ or Hg2+) in extrarenal tissues and then accumulates in the proximal tubule region of the nephron via mechanism of filtration–reabsorption as conjugates of glutathione with Hg^5,6^; causing kidney damage^7,8^.

Once accumulated in the kidney proximal tubule cells, inorganic Hg interrupts intracellular homeostasis by inducing oxidative stress, subcellular organelle dysfunction, and apoptosis as a consequence of reactive oxygen species (ROS) generation, which leads to kidney injury^9–11^. The Hg has an affinity for sulfhydryl groups (–SH) such as those found in glutathione, the most abundant non-protein thiol-containing compound in the cell, thereby mitigating its toxicity and maintaining cellular redox status^12,13^. Therefore, glutathione-related enzymes also play key roles in the natural detoxification process of Hg by controlling the glutathione levels^14,15^ and conjugates of glutathione with Hg^16^.

Currently, genetic variation is considered an important contributor to heavy metal body retention and metabolism^17^. Several epidemiological studies have suggested that genetic variants in glutathione-related genes may significantly influence Hg toxicokinetics, especially some single nucleotide polymorphisms (SNPs) in some glutathione-related enzymes which have been associated with blood and urine Hg concentrations^6,18,19^. However, the gene-Hg interactions have not been reported in relation to kidney injury, which needs to be addressed in order to understand the impact of genetic variation on health effects caused by this heavy metal. In this context, we carried out an epidemiological study on occupational exposure to Hg vapor on kidney function in a historically gold-mining town in Colombia. In this study, we reported that, despite higher levels of Hg in blood and urine in miners compared to a control group, the kidney function was normal and comparable between both groups^20^. This is especially interesting since it has been reported that SNPs in detoxification genes are associated with toxic metal tolerance and adaptation in humans^21^. Therefore, we hypothesized that genetic variants in glutathione-related genes could modulate the negative impact of Hg on the kidneys.

The aim of the current work was to investigate whether single nucleotide polymorphisms (SNPs) in the genes glutamate-cysteine ligase catalytic subunit (*GCLC*); glutamate-cysteine ligase modifier subunit (*GCLM*); glutathione synthetase (*GSS*); glutathione *S*-transferase alpha 1 (*GSTA1*); and glutathione S-transferase pi 1 (*GSTP1*) are associated with modulation of Hg nephrotoxicity in our gold mining population in Colombia.

## Results

### Study population

Sociodemographic characteristics of the study participants and measurement of biomarkers of Hg exposure and effect are presented in Table 1. All loci were in Hardy–Weinberg equilibrium in the study control group. The genotype and allele frequencies are reported in Table 2. According to the hierarchy AMOVA, no genetic structure was evidenced (F_ST_ = 0.00521; *P* = 0.10182 ± 0.00091) between the exposed and non-exposed groups.

**Table 1:** Sociodemographic characteristics, exposure and effect biomarkers of study population.

Variable	Exposure	Non-exposure	*P *value
	N = 160	N = 121	
	n (%) or median (IQR)	n (%) or median (IQR)	
Male^a^	101 (63.12)	59 (48.76)	0.022
Female^a^	59 (36.88)	62 (51.24)	
Age (years)^b^	40 (18–62)	47 (22–59)	< 0.001
Blood-Hg (μg Hg/L)^b^	7.0 (3.4–11.0)	2.5 (2.5–4.7)	< 0.001
Urine-Hg (μg Hg/g creatinine)^b^	3.8 (2.9–10.1)	2.9 (2.9–3.0)	< 0.001
Hair-Hg (μg Hg/g hair)^b^	0.8 (0.5–1.3)	0.4 (0.2–0.7)	< 0.001
eGFR (mL/min/1.73 m^2^)^b^	82.6 (74.5–89.6)	75.7 (69.3–84.1)	< 0.001
Urinary albumin (mg/24 h)^b^	64.1 (48.6–94.6)	87.9 (54.1–132.7)	< 0.001
β-2MCG (ng/mL)^b^	41.1 (23.1–62.9)	36.6 (22.3–64.6)	0.988

*Hg* mercury, *β-2MCG* β-2-microglobulin, *eGFR* estimated glomerular filtration rate, *IQR* interquartile range.

^a^Pearson’s Chi-square test.

^b^Mann–Whitney U test/Wilconxon rank-sum test for differences between groups.

**Table 2:** Distribution of genetic variants in glutathione-related genes in Hg exposed and non-exposed groups.

Gene	SNP	Genotype/allele	Exposed		Non-exposed		HWE *P* value	*P *value^d^	*P* value^e^	OR (95% CI)
			N = 160		N = 121					
			N (%)		N (%)					
*GCLC*	rs1555903	TT	66 (41.3)		48 (39.7)		0.96^a^	0.96	0.81	
		TC	73 (45.6)		56 (46.3)		0.94^b^		1.0	
		CC	21 (13.1)		17 (14.0)		0.94^c^		0.85	
		T	205 (64.0)		152 (62.8)					
		C	115 (36.0)		90 (37.2)			0.78		1.05 (0.75–1.49)
*GCLM*	rs41303970	CC	63 (39.4)		72 (59.5)		0.31^a^	**0.0015**	**0.0009**	0.44 (0.27–0.72)
		CT	80 (50.0)		45 (37.2)		0.45^b^		**0.039**	1.69 (1.04–2.73)
		TT	17 (10.6)		4 (3.3)		0.32^c^		**0.026**	3.48 (1.13–10.6)
		C	206 (64.3)		189 (78.0)					
		T	114 (35.7)		53 (22.0)			**0.0004**		1.97 (1.35–2.89)
*GSS*	rs3761144	CC	64 (40.0)		53 (43.8)		0.047^a^	0.43		
		CG	64 (40.0)		51 (42.2)		0.49^b^			
		GG	32 (20.0)		17 (14.0)		0.036^c^			
		C	192 (60.0)		157 (64.8)					
		G	128 (40.0)		85 (35.2)			0.23		1.23 (0.87–1.74)
*GSTA1*	rs3957356	CC	71 (44.4)		45 (40.2)		0.71^a^	0.18		
		CT	69 (43.1)		59 (52.7)		0.07^b^			
		TT	20 (12.5)		8 (7.1)		0.45^c^			
		C	211 (66.0)		149 (66.5)					
		T	109 (34.0)		75 (33.5)			0.92		1.03 (0.71–1.47)
*GSTP1*	rs4147581	CC	76 (47.5)		43 (35.5)		0.62^a^	**0.015**	**0.042**	1.64 (1.01–2.66)
		CG	71 (44.4)		55 (45.4)		0.56^b^		0.89	
		GG	13 (8.1)		23 (19.1)		0.84^c^		**0.012**	0.37 (0.18–0.77)
		C	223 (69.7)		141 (58.3)					
		G	97 (30.3)		101 (41.7)			**0.005**		0.60 (0.43–0.86)

Statistically significant association are shown in bold (P value < 0.05).

The statistical significance was determined using Pearson’s Chi-square test with P value simulated from 2000 permutations.

P value for Hardy–Weinberg equilibrium in exposed^a^ and non-exposed^b^ groups, and all samples^c^.

^d^*P *value for allele and genotype associations.

^e^*P* value for each genotype versus the other two genotypes.

### Association of genetic variants with Hg levels and kidney function biomarkers

Statistically significant differences in the distribution of genotypes and alleles of *GCLM* rs41303970 and *GSTP1* rs4147581 were observed between the groups. Individuals in the exposed group had an over-representation of the rs41303970-T allele (OR = 1.97; *P* = 0.0004) but an under-representation of the rs4147581-G allele (OR = 0.60; *P* = 0.005) compared with the non-exposed group (Table 2). No other statistically significant differences were found in frequency distribution between these two groups.

**Figure 1:**
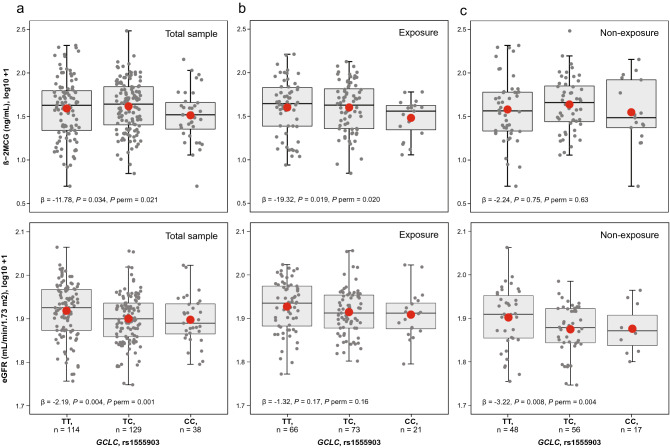
Beta-2-microglobulin (β-2MCG) and estimated glomerular filtration rate (eGFR) in relation to *GCLC* rs1555903 genotypes. The boxplot shows a significantly decreasing trend of both β-2MCG and eGFR in the total sample (a, *P* < 0.05). Analysis by exposure status also shows a similar trend in the exposure group with β-2MCG (**b, **β = − 19.32, *P* = 0.019, *P* perm = 0.020) and in the non-exposure group with eGFR (**c**, β = − 3.22, *P* = 0.008, *P* perm = 0.004). Each SNP was examined using multivariable linear regression models assuming additive model of inheritance adjusted by age, sex, blood-Hg levels (µg Hg/L), urine-Hg levels (µg Hg/g creatinine), hair-Hg levels (µg Hg/g hair), and duration of exposure (years).

A multivariable linear regression model was used to test the effects of SNPs on Hg levels by an additive model. In the combined sample there was a statistically significantly increased urine-Hg (β = 0.062; *P* = 0.047 and *P* perm = 0.009), but not blood-Hg (*P* = 0.63) in relation to the *GCLM* rs41303970-T allele, adjusting by exposure status, sex, age, estimated glomerular filtration rate (eGFR), and hair-Hg levels (Table 3). With respect to biomarkers of renal injury, we found statistically significant associations in relation to the *GCLC* rs1555903-C allele by lowering the levels of eGFR and beta-2-microglobulin (β-2MCG) in the non-exposed group (β = − 3.22; *P* = 0.008, *P* perm = 0.004) and the exposed group (β = − 19.32; *P* = 0.02, *P* perm = 0.02), respectively (Table 4, Fig. 1).

**Table 3:** Association of genetic variants in glutathione-related genes with Hg levels in blood and urine.

Gene	SNP	N	Blood-Hg (µg Hg/L)			Urine-Hg (µg Hg/g creatinine)		
			β	*P* value^a^	*P* value^b^	β	*P* value^a^	*P* value^b^
*GCLC*	rs1555903	281	− 0.042	0.07	0.03	− 0.008	0.76	0.74
*GCLM*	rs41303970	281	− 0.012	0.65	0.56	**0.062**	**0.047**	**0.009**
*GSS*	rs3761144	281	0.014	0.51	0.55	− 0.002	0.94	1.0
*GSTA1*	rs3957356	272	0.032	0.19	0.74	− 0.012	0.67	0.32
*GSTP1*	rs4147581	281	0.004	0.86	0.88	− 0.003	0.92	1.0

Statistically significant association are shown in bold (P value < 0.05).

Each SNP was examined using multivariable linear regression models assuming additive models of inheritance adjusted by exposure status, age, sex, eGFR, and hair-Hg levels (µg Hg/g hair).

^a^*P* value from a generalized linear model.

^b^Empirical *P* value determined based on 5000 permutations from a generalized linear model.

**Table 4:** Association of genetic variants in glutathione-related genes with kidney function biomarkers.

Gene	SNP	Biomarker	Total sample N = 272			Exposure N = 155			Non-exposure N = 117		
			β	*P* value^a^	*P* value^b^	β	*P* value^a^	*P* value^b^	β	*P* value^a^	*P* value^b^
*GCLC*	rs1555903	eGFR	**− 2.19**	**0.004**	**0.001**	− 1.32	0.17	0.16	**− 3.22**	**0.008**	**0.004**
		Albumin	− 48.18	0.15	0.12	− 68.59	0.25	0.10	− 21.71	0.06	0.04
		β-2MCG	**− 11.78**	**0.034**	**0.021**	**− 19.32**	**0.02**	**0.02**	− 2.24	0.75	0.63
*GCLM*	rs41303970	eGFR	− 1.54	0.085	0.07	− 0.73	0.49	0.39	− 3.15	0.05	0.07
		Albumin	− 52.49	0.18	0.11	− 71.46	0.28	0.17	− 21.42	0.15	0.09
		β-2MCG	2.78	0.67	0.96	2.94	0.74	1.0	2.85	0.75	1.0
*GSS*	rs3761144	eGFR	− 0.03	0.97	1.0	− 0.26	0.76	0.94	0.28	0.82	0.82
		Albumin	55.39	0.08	0.05	81.47	0.13	0.07	15.65	0.18	0.54
		β-2MCG	− 3.41	0.51	0.58	− 1.77	0.81	1.0	− 4.41	0.54	1.0
*GSTA1*	rs3957356	eGFR	0.95	0.23	0.12	0.54	0.58	0.60	1.78	0.21	0.19
		Albumin	66.02	0.07	0.05	103.89	0.08	0.14	− 9.64	0.49	0.57
		β-2MCG	6.57	0.26	0.21	7.89	0.33	0.14	0.51	0.95	0.96
*GSTP1*	rs4147581	eGFR	0.32	0.77	0.66	0.72	0.48	0.96	− 0.12	0.92	0.91
		Albumin	− 36.53	0.29	1.0	− 64.89	0.31	0.68	− 7.33	0.54	0.62
		β-2MCG	− 1.82	0.74	1.0	− 11.26	0.19	0.09	8.53	0.24	0.61

Statistically significant association are shown in bold (P value < 0.05).

Each SNP was examined using multivariable linear regression models assuming additive model of inheritance adjusted by age, sex, blood-Hg levels (µg Hg/L), urine-Hg levels (µg Hg/g creatinine), hair-Hg levels (µg Hg/g hair), and duration of exposure (years). Having lower eGFR levels, higher albumin levels or higher β-2MCG levels are indicative of kidney injury.

^a^*P* value from a generalized linear model.

^b^Empirical *P* value determined based on 5000 permutations from a generalized linear model.

Considering that sex-differences in the toxic effect of chemicals that people are exposed to in the working and general environment are to be expected^22^, we assessed the effect modification by sex for the association with kidney function of the *GCLC* rs1555903 and *GCLM* rs41303970 SNPs. While sex showed statistically significant difference in the study population (Table 1), there was no evidence of effect modification by sex for this genetic variant (*P* > 0.7 for eGFR and *P* > 0.3 for β-2MCG). In our multivariable linear regression model, the *GCLC* rs1555903 SNP remained statistically significantly associated with the biomarkers of renal injury, but the magnitude of the regression coefficient decreased by 9.8% with eGFR (β value from − 2.43 to − 2.19) and by 7.5% with β-2MCG (β value from − 20.88 to − 19.32) after adjustment for sex.

### Interaction effects of genetic variants on Hg levels and kidney function biomarkers

**Figure 2:**
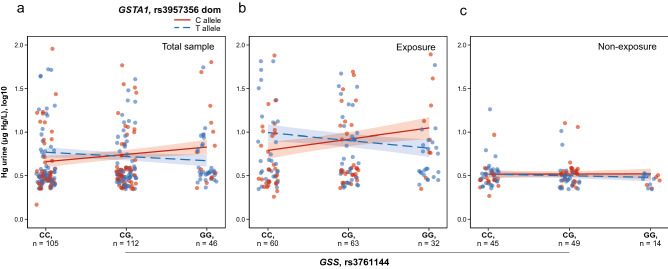
Interaction effects of genetic variants in a multivariable model of urine-Hg. The urine-Hg concentration was defined as a function of the interaction between *GSS* rs3761144 under an additive model and *GSTA1* rs3857356 under a dominant model. The interaction plot shows a significantly increasing trend of urine-Hg levels when individuals carry both rs3761144-G and rs3957356-C alleles (*P* = 0.015, *P* perm = 0.017) in the total sample (**a**). A simple slope analysis showed that the association between urine-Hg and *GSS* rs3761144 was stronger in individuals with rs3957356-C allele compared to individuals with rs3957356-T allele in the exposed group (β = 0.13, *P* = 0.04) (**b**) but not in the non-exposed group (**c**). All interaction analyses were adjusted by age, sex, eGFR, and hair-Hg levels (µg Hg/g hair). Total sample was also adjusted by exposure status.

To identify epistasis between genetic loci, we used a gene interaction analysis between all pairs of individual SNPs by generalized linear regression models. We identified a significant epistatic interaction between *GSS* rs3761144 and *GSTA1* rs3957356 on urine-Hg levels (*P* = 0.015, *P* perm = 0.017). A simple slope analysis showed that the effect of the rs3761144-G allele on the increase in urine-Hg levels only exists when individuals carry the rs3957356-C allele in the exposed group (β = 0.13, *P* = 0.04) but not in the non-exposed group (β = 0.01, *P* = 0.95) (Fig. 2). No significant interactions were found with any of the kidney function biomarkers assessed.

## Discussion

In the kidneys, inorganic Hg species accumulate mainly in the proximal tubule cells, causing renal injury by oxidative stress mechanisms^9^. While glutathione is certainly an important physiological antioxidant of Hg, the glutathione-Hg conjugates might also contribute to intracellular retention of Hg since these conjugates may not be transported easily out of the proximal tubule cells^23,24^. Our study suggests that SNPs in glutathione-related genes might influence toxic effects of Hg by modulating glutathione concentrations in individuals occupationally exposed to this heavy metal.

Studies have shown that GCL is the main determinant of cellular glutathione levels since it catalyzes the first and rate-limiting step in glutathione synthesis^25^. Glutathione de novo synthesis is thought to be induced primarily by transcriptional regulation^26,27^. In this regard, the SNPs *GCLM* rs41303970 and *GCLC* rs1555903, located in the 5′-flanking regions of their respective genes, might affect gene expression at a transcriptional level. On the one hand, the C-to-T substitution at *GCLM* rs41303970 leads to less GCLM enzyme production^28^. We also found that the T-allele of this SNP was associated with higher urine-Hg levels (Table 3), which is in agreement with previous reports on mining settings^6,29^. On the other hand, the T-to-C substitution at *GCLC* rs1555903 leads to higher *GCLC* gene expression, as shown in a small set of kidney cortex samples (N = 73, β = 0.73, *P* = 0.008) from the Genotype-Tissue Expression (GTEx) dataset^30^. A study reported that the rs1555903-C allele is associated with methyl-Hg retention in the umbilical cord^31^. We did not find an association between this SNP and blood- or urine-Hg levels (Table 3). However, we found that the C-allele of this SNP was associated with lower levels of β-2MCG in the exposed group but not in the non-exposed group (Table 4, Fig. 1), suggesting a better performance of the kidney tubular function.

**Figure 3:**
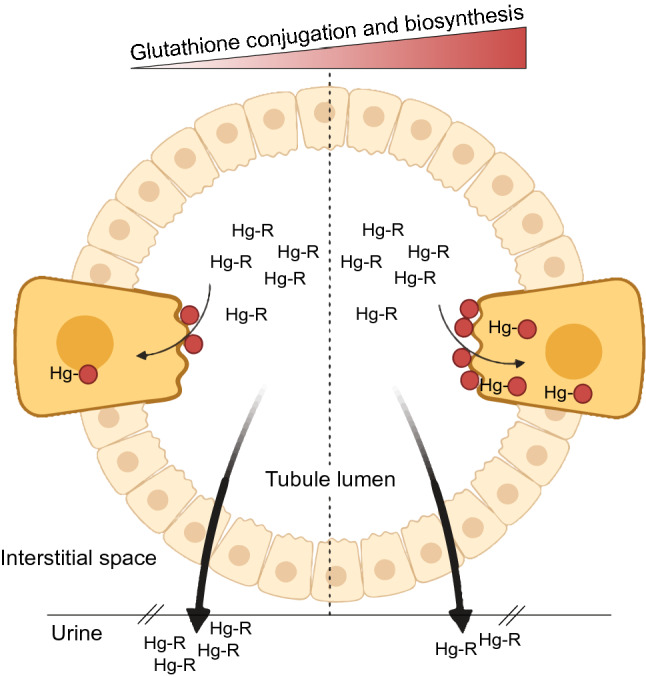
Graphical representation of contributing biosynthesis and conjugation of glutathione underlying Hg-kidney toxicity. The graph shows a cross-section of the proximal tubule of neprhon, where two cells were magnified to represent the reabsorption of glutathione-Hg conjugates. Hg contained in the primary urine after glomerular filtration is presented as Hg-R, where R is any molecule attached to it (e.g., albumin). The dotted line split the graph in two sections according to the level of biosynthesis and conjugation of glutathione (red circles): lower levels on the left and high levels on the right. At the level of the luminal plasma membrane of proximal tubular epithelial cells, glutathione-Hg conjugates are internalized by a mechanism of molecular homology such as dicysteinylmercury (cysteine-Hg-cysteine). Thus, decreasing both biosynthesis (as in *GCLM* rs41303970-T carriers) and/or conjugation (as in *GSTP1* rs4147581-C carriers) of glutathione could increase the Hg elimination in the urine due to a lower rate in the uptake of glutathione-Hg conjugates, mitigating kidney injury by this heavy metal. Created with BioRender.com and edited with Adobe Illustrator.

While GCLC and GCLM are the subunits that constitute the GCL holoenzyme, they are not necessarily present in equimolar amounts within the cell^27^. GCLM interacts with GCLC, making the GCL holoenzyme kinetically more efficient in glutathione synthesis since GCLC has a very high Km for l-glutamate compared to when it is complexed with GCLM^32,33^. In this context, a low expression level of *GCLM* due to the rs41303970-T allele might result in a low synthesis rate of glutathione and hence the formation of Hg-glutathione conjugates^6^. At the kidney level, uptake of glutathione-Hg conjugates occurs mainly at the luminal plasma membrane of proximal tubular epithelial cells^12,34^. In Vivo findings have suggested that this process depends greatly on the actions of both γ-glutamyltransferase and cysteinylglycinase, which form conjugates of l-cysteine (*e.g.*, dicysteinylmercury) that are internalized through amino acid transporters via a mechanism of molecular homology^9,34^. Therefore, a reduction in the formation of Hg-glutathione conjugates minimizes its uptake rate into the kidneys after glomerular filtration of Hg contained in the primary urine, thus favoring the excretion of Hg (Fig. 3).

In the same direction, we found that the rs4147581-C allele in the glutathione conjugation gene *GSTP1* is significantly more frequent in the exposed than in the non-exposed group (Table 2). This allele has been associated with DNA methylation^35^, which might be down-regulating the *GSTP1* gene expression^36^. These data could explain, at least in part, why we found lower eGFR levels in relation to the *GCLC* rs1555903 SNP in the non-exposed but not in the exposed group (Table 4). Thus, having the alleles *GCLC* rs1555903-C and *GCLM* rs41303970-C drives to more glutathione production, which could be efficiently conjugated to Hg by members of the GST-superfamily such GSTP1—the most widely expressed GST enzyme according to the Human Protein Atlas^37^, including in the kidneys. The *GSTP1* rs4147581 is in linkage disequilibrium with rs1695 (D′ = 0.99, R^2^ = 0.44 in Admixed American populations, including Colombians), a common nonsynonymous SNP that changes the 105th amino acid from isoleucine to valine, altering the substrate binding site of GSTP1^38^. It has been reported that the GSTP1 Val105 not only has a significantly lower affinity for glutathione, but also is more sensitive to inhibition by Hg species^38^. Since the GSTP1 Val105 correlates with rs4147581-C allele (Chi-square = 63.17, *P* < 0.0001) in Colombians from the 1000 Genomes Project^39^ as calculated in the LDlink web-based application^40^, these genetic variants could be conferring some protection to the exposed group by negatively affecting the formation of Hg-glutathione conjugates (Fig. 3).

Considering that gene–gene interactions may contribute to inter-individual variation in complex traits^41,42^, we tested whether epistatic interactions for SNP pairs can contribute to regulating Hg body retention and nephrotoxicity. We identified a significant SNP-SNP interaction between the *GSS* rs3761144-G and *GSTA1* rs3957356-C alleles with a clearance of urine-Hg at a higher rate in the exposed but not in the non-exposed group (Fig. 2). Both alleles, located in the 5′-flanking regions, down-regulate the expression of their respective genes^43,44^. In this context, low expression of these two genes would lead not only to decreased glutathione synthesis by the GSS enzyme, but also to decreased glutathione conjugation by the GSTA1 enzyme^16,45^. It has been reported that the rs3761144-G allele is associated with higher levels of hair methyl-Hg due to fish consumption^44^, but the rs3957356-C allele did not show a similar trend with levels of methyl-Hg in erythrocytes^18^.

Taken together, these pieces of evidence suggest that the biosynthesis rate of glutathione might be involved in nephrotoxicity by increasing cellular retention of Hg, altering the redox balance and leading to cytotoxicity^46,47^, possibly via mitochondria dysfunction by altering the ratio of reduced (GSH) to oxidized (GSSG) glutathione in this organelle^48,49^. Therefore, decreasing both biosynthesis and conjugation of glutathione could positively impact the body’s ability to clear Hg. This is supported by the observations that: (1) inorganic Hg accumulation in the proximal tubule region of the nephron is mainly handled by filtration–reabsorption as glutathione-Hg conjugates^5,6^, and (2) the renal uptake of glutathione-Hg conjugates occurs mainly at the luminal membrane via a molecular mimicry mechanism using animo acid transporters^9,12^. The study population offers some unique opportunity to explore whether genes can provide protection to a specific disease-related environmental exposure. This is because, contrary to expectations, we did not find meaningful associations between occupational Hg vapor exposure and altered kidney function monitoring parameters^20^. It is important to mention that both the exposed and the non-exposed groups are not a migrant population but a stationary population. Indeed, they have been living for several generations in those towns (i.e., since Colonial times), where historically they have practiced the same economic activity^50^. This population feature helped us to capture some inherited genetic differences that might be modifying the toxic effect of chronic Hg exposure. In this regard, in humans, it has been suggested that an increase in the frequencies of protective variants in detoxification genes might be due to a mechanism of adaptation to toxic environmental metal tolerance^21^.

A few remarks can be made regarding the limitations of this study. While the study included a small sample size, it represented almost the entire non-genetically structured population from the mining and non-mining communities. We selected SNPs in glutathione-related genes based on their reported involvement in Hg metabolism. However, other SNPs in linkage disequilibrium or otherwise might also be influencing the glutathione-Hg metabolism. In addition, since multiple factors underlie the complex pathogenesis of Hg toxicity including genetic and environmental factors, their interactions could also be contributing to the associations found in our study. Hopefully, similar studies in well-characterized cohorts might ultimately enlighten the role of glutathione-related genetic variants in Hg toxicity (e.g., via meta-analysis).

Our genetic epidemiological findings suggest that in the historically artisanal and small‐scale gold mining study population, the genetic variants analyzed in the glutathione-related genes could modulate the pathogenesis of Hg nephrotoxicity by controlling the glutathione concentrations. Replication studies for genetic variants in these and in other glutathione-related genes are necessary to clarify the role of glutathione in Hg toxicity.

## Methods

### Study design, population, and sample collection

A cross-sectional study was performed in mining and non-mining groups with similar socio-demographic characteristics from northeastern Colombia. In an unmatched population-based case–control approach, we estimated a sample size of 258 individuals with QUANTO software, version 1.2.4, assuming a log-additive inheritance model at 5% significance and 80% power and using the following parameters based on our previous study^20^: an environmental exposure to Hg of 2.5%, a population risk of reduced estimated glomerular filtration rate (eGFR; < 76.4 mL/min per 1.73 m^2^) of 25%, an environmental effect of reduced eGFR per tenfold increase in blood Hg level of 1.3, an estimated genetic effect of 1.8, an estimated gene-environment interaction effect of 1.2, and a minor allele frequency of 0.2 using data of Colombian-ancestry individuals (CLM) from the 1000 Genomes Project^39^.

The study population was comprised of 281 participants: 160 in the exposed and 121 in the non-exposed group. Similar socio-demographic characteristics were found in the two communities (Table 1). Both clinical and epidemiological information was collected for each participant through a detailed personal interview. An excess of heterozygosity was found (FIS = − 0.0530, *P* = 0.96), indicating an absence of endogamy in this population. All participants were 18–62 years old and provided informed consent to participate in the study. Individuals in the exposed group have been residents in the gold mining districts for at least the last 5 years prior to the study and had direct contact with Hg vapors in the last year. Individuals in the non-exposed group were permanent residents of non-mining towns and had no life history of direct contact with Hg vapors. All relevant information about study design, population, and sample collection characteristics was previously described in detail^51^.

### Quantification of mercury and kidney function biomarkers

We utilized data from our previously published studies^20,51^, which detail methods and analyses. Briefly, total blood-Hg and urine-Hg were measured using a S4 atomic absorption spectrometer equipped with a VP100 hydride generation system (Thermo Electron Co., Cambridge, UK), whereas total hair-Hg concentration was quantified using an RA-915+ atomic absorption spectrometer mercury analyzer with Zeeman background correction and coupled to a RP-91C pyrolysis chamber (Lumex, St. Petersburg, Russia)^51^. Total Hg was used as biomonitoring data since it can give precise information on the total internal exposure of an individual at a given point in time, whereas total hair-mercury was used as a confounder for the effects of methyl-Hg due to fish/seafood consumption^52,53^. We reported medians and interquartile ranges for concentrations of total Hg in the exposed group and the non-exposed group (Table 1)^51^. Testing was carried out at the Industrial Consultation Laboratory of the Universidad Industrial de Santander that has been accredited according to ISO/IEC 17025:2005, performing well in international quality control programs.

The glomerular function was evaluated by determining serum and 24-h urine creatinine, urinary albumin in the first-morning sample, and by estimating glomerular filtration rate (eGFR) with the CKD-EPI formula^54^. Creatinine was measured by spectrophotometry using the Selectra JR Clinical Chemistry Analyser (Vital Scientific, France). Urinary albumin was measured by a competitive immunoassay using the Siemens Immulite One analyzer (Siemens Healthcare Diagnostics, Germany). The tubular function was evaluated by determining urinary excretion of the beta-2-microglobulin (β-2MCG) by an immunometric assay using the Siemens Immulite One analyzer (Siemens Healthcare Diagnostics, Germany)^20^.

### DNA isolation and genotyping

Total genomic DNA was extracted from 5 ml of EDTA-treated peripheral whole blood using the standard salting-out method^55^. DNA concentration was determined with a Nanodrop One Spectrophotometer (Thermo Fisher Scientific, USA) and adjusted to 20 ng/μL with TE buffer. The SNPs glutamate-cysteine ligase catalytic subunit, *GCLC*, rs1555903; glutamate-cysteine ligase modifier subunit, *GCLM*, rs41303970; glutathione synthetase, *GSS*, rs3761144; glutathione *S*-transferase alpha 1, *GSTA1*, rs3957356; and glutathione S-transferase pi 1, *GSTP1*, rs4147581 were genotyped by TaqMan-based allelic discrimination assay (Applied Biosystems, USA) using a CFX96 Touch Real-Time PCR detection system (Bio-Rad, USA).

### Statistical analysis

To determine the presence of population genetic structure, an analysis of molecular variance (AMOVA) was performed with Arlequin, version 3.5^56^. The differences between the genotypic and allelic frequencies of the study groups were compared by the Pearson Chi-square test, and odds ratios (OR) were established with a 95% confidence interval (95% CI). The Fisher’s exact test was used when the genotypic and allelic frequencies were less than 5%. Multivariable generalized linear regression models were used to assess the effects of SNPs on Hg levels and on kidney function biomarkers: eGFR, urinary albumin, and β-2MCG while adjusting for possible confounders such as sex and age as well as hair-Hg levels and duration of exposure to minimize the overestimation of Hg^0^ exposure^57,58^. Each SNP was coded as 0, 1 or 2 according to the count of their minor allele. *P* values less than 0.05 (*P* < 0.05) were considered statistically significant. The nominal significance level was retained when significant empirical *P* values were obtained through 5000 replicate permutations^59,60^. Interactions between SNPs were examined by logistic regression to analyze their combined effects on Hg levels and kidney function biomarkers. The significant interaction terms were decomposed by a simple slope analysis. Statistical analyses were performed with R programming language, version 3.5.1.

### Ethics approval and consent to participate

The study was conducted under protocols approved by the Scientific Research Committee of the Universidad Industrial de Santander and complied with the Colombian Medical Code of Ethics, which is in accordance with the ethical standards laid down in the 1964 Declaration of Helsinki.

## Data Availability

The data that support the findings of this study are available from the corresponding author upon reasonable request. The data are not publicly available due to the nature of the questions asked in this study, them containing information that could compromise research participant consent.
